# Genetic Architecture of Skewed X Inactivation in the Laboratory Mouse

**DOI:** 10.1371/journal.pgen.1003853

**Published:** 2013-10-03

**Authors:** John D. Calaway, Alan B. Lenarcic, John P. Didion, Jeremy R. Wang, Jeremy B. Searle, Leonard McMillan, William Valdar, Fernando Pardo-Manuel de Villena

**Affiliations:** 1 Department of Genetics, University of North Carolina, Chapel Hill, North Carolina, United States of America; 2 Lineberger Comprehensive Cancer Center, University of North Carolina, Chapel Hill, North Carolina, United States of America; 3 Carolina Center for Genome Sciences, University of North Carolina, Chapel Hill, North Carolina, United States of America; 4 Curriculum in Genetics and Molecular Biology, University of North Carolina, Chapel Hill, North Carolina, United States of America; 5 Department of Computer Science, University of North Carolina, Chapel Hill, North Carolina, United States of America; 6 Department of Ecology and Evolutionary Biology, Cornell University, Ithaca, New York, United States of America; University of Wisconsin–Madison, United States of America

## Abstract

X chromosome inactivation (XCI) is the mammalian mechanism of dosage compensation that balances X-linked gene expression between the sexes. Early during female development, each cell of the embryo proper independently inactivates one of its two parental X-chromosomes. In mice, the choice of which X chromosome is inactivated is affected by the genotype of a *cis*-acting locus, the *X-chromosome controlling element* (*Xce*). *Xce* has been localized to a 1.9 Mb interval within the X-inactivation center (*Xic*), yet its molecular identity and mechanism of action remain unknown. We combined genotype and sequence data for mouse stocks with detailed phenotyping of ten inbred strains and with the development of a statistical model that incorporates phenotyping data from multiple sources to disentangle sources of XCI phenotypic variance in natural female populations on X inactivation. We have reduced the *Xce* candidate 10-fold to a 176 kb region located approximately 500 kb proximal to *Xist*. We propose that structural variation in this interval explains the presence of multiple functional *Xce* alleles in the genus *Mus*. We have identified a new allele, *Xce^e^* present in *Mus musculus* and a possible sixth functional allele in *Mus spicilegus*. We have also confirmed a parent-of-origin effect on X inactivation choice and provide evidence that maternal inheritance magnifies the skewing associated with strong *Xce* alleles. Based on the phylogenetic analysis of 155 laboratory strains and wild mice we conclude that *Xce^a^* is either a derived allele that arose concurrently with the domestication of fancy mice but prior the derivation of most classical inbred strains or a rare allele in the wild. Furthermore, we have found that despite the presence of multiple haplotypes in the wild *Mus musculus domesticus* has only one functional *Xce* allele, *Xce^b^*. Lastly, we conclude that each mouse taxa examined has a different functional *Xce* allele.

## Author Summary

Although mammalian females have two X chromosomes in each cell, only one is functional, while gene expression from the other is silenced through a process called X chromosome inactivation. Little is known about the early stages of this process including how one parental X chromosome is inactivated over the other on a cell-by-cell basis. It has been shown, however, that certain inbred mouse strains are functionally different at a locus that controls this choice that provides an opportunity to identify the locus and determine its molecular mechanism. This has been the goal of many researchers over the past 40 years with incremental success. Here we took advantage of new mouse genotype and whole genome sequencing data to pinpoint the locus controlling choice. Our results identified a smaller region on the X chromosome that contains large duplicated sequences. We propose an explanation for multiple functional alleles in mouse and provide insight into the possible molecular mechanism of X chromosome inactivation choice. Our evolutionary analysis reveals why functional diversity at this locus appears to be common in laboratory mice and offers an explanation as to why we do not see this level of diversity in humans.

## Introduction

The eutherian female is a mosaic of two cell populations that have either a transcriptionally active maternal or paternal chromosome X. This is a consequence of the mammalian dosage compensation mechanism called X chromosome inactivation (XCI) that balances X-linked gene expression between the sexes [1]. The choice of which X chromosome undergoes XCI occurs early during female embryogenesis on a small population of epiblast cells within the embryo proper [2], [3], [4], [5]. By an unknown mechanism, each cell randomly chooses to inactivate one of the two parental X-chromosomes and then commits to that choice by initiating a cascade of transcriptional and epigenetic regulation that modifies both chromosomes to distinguish the future inactive X from the active X [6], [7], [8], [9], [10], [11]. Ultimately, the inactive X chromosome becomes physically condensed and sequestered within the nucleus rendering it almost completely nonfunctional [12], [13], [14]. The initial choice each epiblast cell makes is preserved and transmitted mitotically to all its daughter cells [15]. As a result, each female is a unique mosaic of somatic cells that express either the maternally or paternally derived X chromosome. The degree of mosaicism (overall ratio and spatial distribution of cells) is determined by the initial number of cells that undergo independent choice, by the developmental fate of each epiblast cell and its multiplication rate.

**Figure 1:**
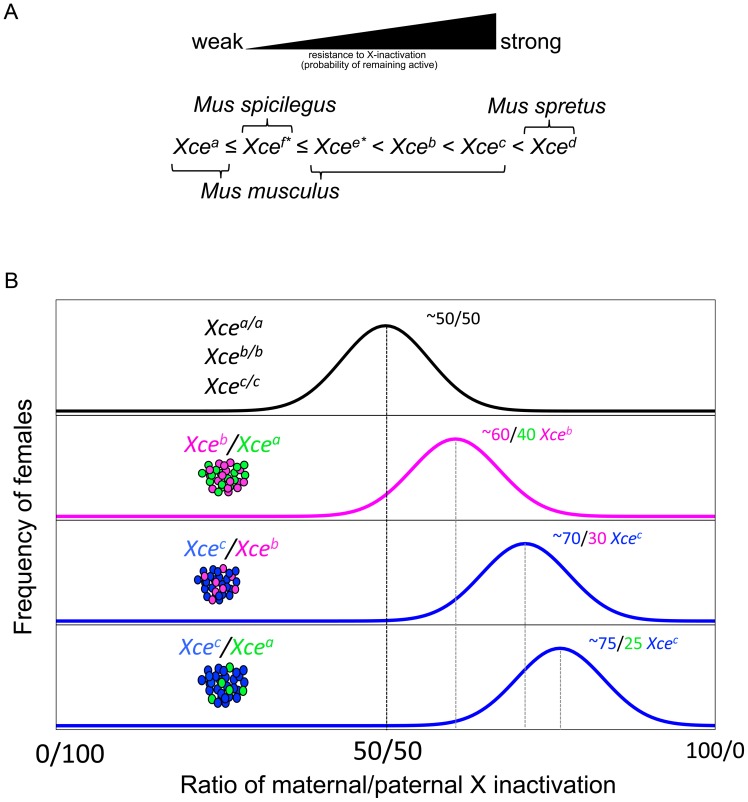
The *Xce* allelic series. Panel A shows the order of *Xce* allele strength. Panel B shows hypothetical distribution and mean XCI ratio skewing in female populations that are either homozygous or heterozygous for *Xce* alleles.

A role for genetics in XCI choice was initially discovered by skewed XCI ratios in female hybrids between certain stocks derived from classical inbred mouse strains. These female hybrids, on average, preferentially inactivated one X chromosome over the other in a strain dependent manner [16], [17]. The effect was later mapped to a single location on the X chromosome and given the name *X-chromosome controlling element* (*Xce*) for its role in XCI choice [18]. Since its initial discovery, four functional alleles of *Xce* have been characterized in *Mus* inbred strains, (*Xce^a^*, *Xce^b^*, *Xce^c^* and *Xce^d^*) and are distinguished by their relative resistance or susceptibility to inactivation [17], [19], [20], [21], 22,23,24. The four *Xce* alleles form an allelic series of XCI skewing, the magnitude and direction of which depends on the *Xce* genotype of the female. Furthermore, XCI skewing is only observed in *Xce* heterozygotes while female homozygotes display no preference towards inactivating either parental X chromosome [25]. The order of known *Xce* allele strength is *Xce^a^ < Xce^b^ < Xce^c^* < *Xce^d^* (Figure 1A). In other words, in female heterozygotes the X chromosome carrying the stronger *Xce* allele has a higher probability of remaining active and thus, these females will have a larger number of cells with that X chromosome active (Figure 1B). From a genetic standpoint, alleles at *Xce* are overdominant and therefore *Xce* acts in *cis*.

*Xce* has been mapped within a 1.85 Mb candidate interval that overlaps with the current definition of the *X inactivation center* (*Xic*) which includes three long non-coding RNAs *Xist*, *Tsix* and *Xite* that play major roles in murine XCI [26]. It has been postulated that the *Xce* allelic series can be explained by genetic variation within these long non-coding RNAs, specifically *Xite* [27]. An alternative hypothesis is that XCI choice is controlled by X-linked and autosomal dosage factors [28], [29], [30] and thus *Xce* would serve as a binding site for a *trans*-acting factor(s) that influences *Tsix* or *Xist* expression [28], [30], [31], [32]. Nonetheless, the identity of *Xce* remains unknown. This is in part due to the technical challenges of measuring XCI choice and to the relatively high level of stochastic variation in XCI in isogenic female populations that together make it difficult to infer with certainty the *Xce* allele present in an individual female (Figure 1B). Mapping *Xce* is further complicated by the comparatively low recombination rate of the X chromosome and the fact that only females are informative for the phenotype.

Although *Xce* is the major locus controlling XCI choice, previous studies have demonstrated that parent-of-origin and autosomal factors significantly influence XCI choice [23], [24], [33], [34], [35]. A large mapping experiment identified suggestive loci on five autosomes but none reached genome–wide significance [26]. The parent-of-origin effect was first described by Forrester and Ansell in 1985 as a difference in XCI skewing depending on whether the *Xce^c^* allele was maternally or paternally inherited in *Xce^c/b^* heterozygotes. The evidence available at the time, however, could not discriminate among *Xce*, another X-linked locus or autosomal loci. A more recent study provided additional evidence of a parent-of-origin effect and postulated that its cause could be *Xce* itself or epigenetic differences of one or more X-linked loci [34]. The same study showed an increased variance in XCI skewing in F2 females heterozygous for the same combination of *Xce* alleles as F1 hybrids, indicating the existence of autosomal factors that influence XCI choice [34]. A more recent study used mouse lines with recombinant X chromosomes derived from two genetically divergent mouse inbred strains (129S1/SvlmJ and CAST/EiJ) to show that multiple regions along the X chromosome influence XCI choice, but was unable to map any of them, including *Xce* [36]. Lastly, there are well-documented cases of secondary XCI skewing that influence the XCI patterns observed in adults [37], [38], [39]. Secondary skewing occurs when an X linked mutation impacts cell survival or proliferation.

Technical issues associated with measuring XCI choice further complicate the identification of *Xce*. A well-established surrogate for XCI choice is X-linked allele-specific gene expression. Nonetheless, gene expression in a female mouse can be influenced by many factors in addition to XCI choice itself. And thus, it is important to carefully choose X-linked genes that most accurately reflect the true ratio of XCI while minimizing the presence of misleading factors such as differential expression due to *cis*-acting regulatory variants, tissue-specific skewing, or XCI escape. As a general rule, estimation of XCI skewing improves with the number of X-linked genes used.

In this study, we developed an approach that overcomes major challenges of mapping *Xce*. Our approach is based on association mapping of XCI skewing phenotypes in classical inbred strains that have recently been genotyped at very high density [40] or had their genome sequenced (whole genome sequence, WGS) [41]. Our analysis was restricted to the previously defined candidate interval [26] and generated a new candidate interval of much smaller size. By generating multiple F1 hybrid females between inbred strains we accurately determined the mean and the variance in XCI ratio within genetically identical mice. We also generated reciprocal crosses to determine the parent-of-origin effects. Lastly, we performed these analyses in multiple tissues and thus determined whether tissue choice had an effect on the estimation of skewing of XCI. In order to analyze the X-linked expression phenotype data we developed a hierarchical Bayesian model and inference procedure that allows to us to estimate both the mean and the variability of XCI within an individual female or female population. We extended our phenotyping to wild-derived inbred strains with different haplotypes of known subspecific origin [40], and used these data to reconstruct the evolutionary history of the *Xce* locus itself.

## Results

### Association mapping based on public data narrows the *Xce* candidate interval to 194 kb

**Figure 2:**
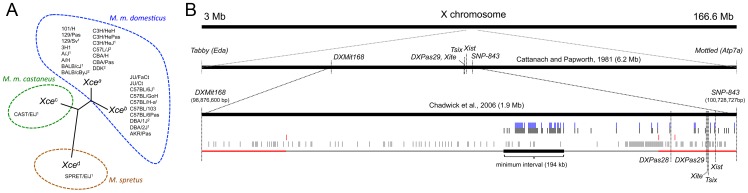
The *Xce* candidate interval based on historical data. Panel A is a phylogenetic tree that reflects the sequence divergence within the Chadwick candidate interval for inbred mouse strains with known *Xce* alleles. Inbred strains with a number one superscript have both MDA and Sanger sequencing information available, while mouse strains with a number two superscript have only MDA genotype data available. Inbred strains with no number are assumed to have identical genotypes to a closely related strain that has been genotyped. Blue and green shading denotes the subspecific origin of the Chadwick interval for each strain (*M. m. domesticus* and *M. m. castaneus*, respectively). Panel B is a physical map that shows the locations of the previous *Xce* candidate intervals [26], [57]. Below the historical candidate intervals are the results of the SDP analyses using inbred strains selected from Panel A (See Methods). Tick marks represent SDPs classified as consistent (black), inconsistent (red), and partially consistent (gray). SNPs that retain consistent SDPs after inclusion of ALS/LtJ, LEWES/EiJ, PERA/EiJ, SJL/J, TIRANO/EiJ, WSB/EiJ, and ZALENDE/EiJ in the analysis are shown as blue tick marks above consistent SDPs. Our new maximum candidate interval is shown in gray below the tick marks. The minimum candidate interval is shown in black, while regions excluded are shown in red.

In our initial approach to reduce the candidate interval we first identified a subset of inbred mouse strains that had both a known *Xce* allele and high-density genotype [40] or sequence data [41] available. Over the past four decades, several inbred mouse strains have been phenotyped for XCI skewing and these strains include representatives of each one of the four known *Xce* alleles (Figure 2A). At the *Xce* candidate interval defined by Chadwick and coworkers (2006), referred hereafter as the Chadwick interval, these strains have haplotypes derived from two different *Mus* species, *Mus spretus* and *Mus musculus*, and two subspecies of the latter, *M. m. castaneus* and *M. m. domesticus* [40]. Two strains, CAST/EiJ and SPRET/EiJ, cannot be used to refine the candidate interval using single locus association mapping techniques because they are singletons for both an *Xce* allele and the specific or subspecific origin (Figure 2A). The remaining 25 strains are almost evenly distributed between *Xce^a^* and *Xce^b^* carriers and all have a *M. m. domesticus* haplotype in the candidate interval [40]. Furthermore, all of them are classical inbred strains descended from a small pool of founders [42] which makes extremely unlikely the possibility that one or more recurring mutations that exactly generate either the *Xce^a^* or the *Xce^b^* allele arose multiple times independently. Thus, it is reasonable to assume that *Xce^a^* and *Xce^b^* alleles are inherited from a recent common ancestor rather than spontaneously arising over multiple times within this complex multifamily pedigree. Eleven of these strains (or a closely related sister strain) have been genotyped at high density and eight have been sequenced [40], [41]. Importantly, both alleles are represented among genotyped and sequenced strains (*Xce^a^*, seven genotyped and five sequenced strains and *Xce^b^*, four genotyped and three sequenced strains, Figure 2A).

For every SNP and indel present within the Chadwick interval, we determined the pattern of allelic similarities and differences among the subset of inbred strains with known *Xce* alleles (Strain Distribution Pattern (SDP), see Materials and Methods and Figure S1) [43], [44]. SDPs were then classified into three categories based on consistency between phenotype and genotype: 1) fully consistent with the *Xce* phenotype (black tick marks), 2) inconsistent with the *Xce* phenotype (red tick marks), or 3) partially consistent (gray tick marks) (Figure 2B and Table S1). We focused our association analysis within the Chadwick interval, which is based on genetic mapping in populations segregating for the *Xce^a^*, *Xce^b^*, and *Xce^c^* alleles.

Analysis of Mouse Diversity Array (MDA, [44]) genotypes and sequence data shows an enrichment of consistent SDPs (eight MDA SNPs, 120 Sanger SNPs and indels) at an 194 kb interval spanning from rs29082048 to Sanger Mouse Genomes Project (SMGP) SNP position at 100,119,750 bp (Table S1). This interval does not contain any inconsistent SNPs. In addition, there are 23 SNPs with consistent SDPs randomly distributed throughout the distal portion of the Chadwick candidate interval (Figure 2B). These SNPs do not cluster and this region is punctuated with inconsistent SNPs.

We conclude that the minimum *Xce* candidate interval is located approximately 558 kb proximal to *Xist* (note that the maximum *Xce* candidate interval based on this analysis spans from inconsistent SMGP-SNP at position 99,091,507 bp to inconsistent SMGP-indel at 100,460,107 bp). Within this candidate interval all phenotyped strains with the *Xce^a^* allele share the same haplotype and all strains with the *Xce^b^* allele share a different haplotype based on MDA genotypes.

### XCI skewing in experimental F1 hybrids derived from inbred strains within unknown *Xce*

Our ability to reduce further the *Xce* candidate interval depended on the number of inbred strains with known *Xce* allele and high-density genotype data available. Ideally we would like to phenotype inbred strains that have *Xce^a^* and *Xce^b^* recombinant haplotypes in the candidate interval. Furthermore, we would like to characterize the *Xce* alleles of additional *M. m. domesticus* strains with haplotypes that are not associated with known *Xce* allele carriers. These strains will provide additional information about *Xce* functional diversity within *M. m. domesticus* and depending on their *Xce* phenotype, may further refine the *Xce* candidate interval. We selected three strains with *Xce^a/b^* recombinant haplotypes ALS/LtJ, SJL/J and WLA/Pas because of their availability and their ability to refine further the new candidate interval. Based on phylogenetic analysis of the new candidate interval (See Methods), we selected six wild-derived inbred strains, PERA/EiJ, TIRANO/EiJ, ZALENDE/EiJ, LEWES/EiJ, and WSB/EiJ to represent each of the major haplotypes present in *M. m. domesticus* (with the exception of *b3* which has only been observed in wild mice). We selected PWK/PhJ to characterize the *Xce* allele in a third *M. musculus* subspecies, *M. m. musculus*. We selected WSB/EiJ and PWK/PhJ because they are wild-derived strains of *M. m. domesticus* and *M. m. musculus* origin, they have available whole genome sequence [41] and they are founder strains in mouse genetic resources such as the Collaborative Cross [45] and Diversity Outbred [46]. Finally, we selected PANCEVO/EiJ to characterize the *Xce* allele present in a third species of mouse, *Mus spicilegus.* A summary of the justification for selecting each mouse strain and the information it provided towards mapping *Xce* is provided in Table S2.

To determine which *Xce* allele is present in each strain, we generated genetically defined F1 female hybrids by crossing the unknown strain to inbred strains with well-characterized *Xce* alleles: *Xce^a^*, A/J and 129S1/SvImJ; *Xce^b^*, C57BL/6J; and *Xce^c^*, CAST/EiJ. To estimate the presence, direction and extent of XCI skewing in each F1 hybrid female, we developed highly quantitative pyrosequencing assays and measured allele-specific X-linked gene expression (see Methods). On average, for each strain with an unknown *Xce* allele, we tested allele-specific expression in 69 F1 females (ranging from 40 to 120 females per strain, Table S3).

To analyze and integrate the X-linked expression data set, we developed a hierarchical Bayesian model and inference procedure. The method is described briefly in the Methods section, and full description will be reported elsewhere. Briefly, our model parameterizes gene-tissue bias and precision, parent-of-origin effects, and genetic background effects (strain) to account for gross sources of uncertainty and error associated with our XCI phenotyping method. This allows us to combine the different gene measurements and tissues from individual females and establish a mean XCI ratio (see Materials and Methods) for a given cross.

**Figure 3:**
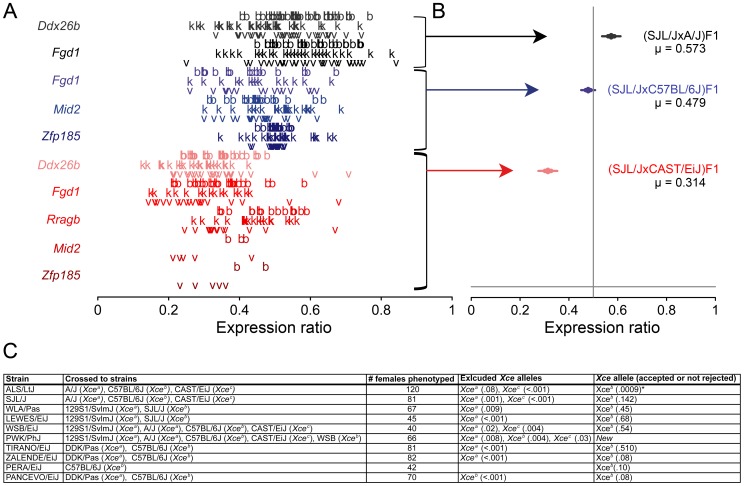
Allelic imbalance in selected female F1 hybrids. Panel A is a plot of the allele-specific expression data from F1 hybrids, where each colored letter represents an individual gene measurement from brain (“b”), kidney (“k”), and liver (“v”) from an individual female. Panel B is a plot of the posterior mean and confidence intervals for XCI fraction inferred for each genetic cross, based on our statistical model. Throughout, the x-axis reports the fraction of X-linked allele-specific expression from the strain with the unknown *Xce* allele. The color of each letter (on the right) and each corresponding posterior (on the left) denote the known *Xce* allele to which it is paired: black *Xce^a^*; blue *Xce^b^* and red *Xce^c^*. Panel C is shows the inbred strains phenotyped for *Xce*, the strains each were crossed to, the total number of F1 females tested and the *Xce* alleles excluded and included based on posterior tail probabilities.

For each F1 cross, we tested whether the two parental strains carry the same *Xce* allele. Figure 3 shows the gene expression data (panel A) and posterior mean and confidence intervals inferred from it (Panel B) for the SJL/J F1 crosses performed. The posteriors in Panel B estimate the mean inactivation proportion associated with each cross. They show where and how posterior probability for the underlying cross mean is concentrated on the scale of 0 (representing full maternal inactivation) to 1 (representing full paternal inactivation), with 0.5 indicating a cross average of about 50% paternal and maternal X-inactivation. By choosing regions of 95% posterior coverage, we see that the data allows us to measure mean X inactivation proportions accurately within 7.7% (+/−5%), placing for instance, the (SJL/JxCAST/EiJ)F1 firmly to the left of 50%, around 33.6% of cells with an active SJL/J X chromosome. As a rule, when a distribution shows a strong bias, in other words, when most of the posterior is concentrated on one side of 0.5 boundary, we use this as evidence to conclude that the two strains involved the cross have functionally different *Xce* alleles. To quantify this bias, we used the tail posterior probability (*i.e*., the amount of posterior probability that lies on the side of 0.5 line, Figure 3C). These tail probabilities are like p-values and their small values strongly support the presence of skewed XCI.

Using this approach, we conclude that seven inbred strains, ALS/LtJ, SJL/J, LEWES/EiJ, PERA/EiJ, TIRANO/EiJ, WSB/EiJ and ZALENDE/EiJ carry an *Xce^b^* allele (Figure 3 and Figure S3). The *M. m. musculus* strain, PWK/PhJ has a new allele, named herein *Xce^e^*. Within the allelic series, the strength of this new allele falls between *Xce^a^* and *Xce^b^* (Figure 1A). Finally, PANCEVO/EiJ has an allele that is similar in strength to *Xce^a^* (Figure S3). The results for the WLA/Pas strain are inconclusive and will be discussed later.

Incorporation of the ALS/LtJ and SJL/J strains to our association mapping further reduced the proximal boundary of the new *Xce* candidate interval by 9.6 kb. Furthermore, by including ALS/LtJ, SJL/J, LEWES/EiJ, PERA/EiJ, TIRANO/EiJ, WSB/EiJ and ZALENDE/EiJ into our SDP analysis, we reduced the number of SNPs with consistent SDPs within the *Xce* interval to 69 and further reduced the proximal boundary by 8.2 kb (Figure 2B, blue tick marks and Table S4). The minimum refined *Xce* candidate interval is bounded by SMGP-SNPs at positions 99,943,259 bp and 100,119,750 bp.

Outside of the refined candidate interval but within the Chadwick interval only 14 SNPs (WGS and MDA data) have consistent SDPs (Table S4). These SNPs (highlighted blue in Figure 2B) do not cluster and are interspersed with SNPs with inconsistent SDPs. Lastly, only three SNPs on the entire X chromosome (rs29079362, rs73483921 and rs29081860) outside of the Chadwick interval have SDP patterns consistent with the *Xce* alleles.

### Analysis of the *Xce* candidate interval reveals a set of segmental duplications associated with each functional *Xce* allele

**Figure 4:**
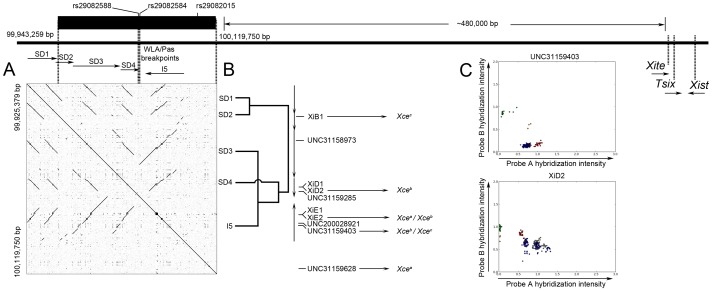
Sequence analysis of the candidate interval. In panel A, the candidate interval is show as a thick black bar. Below the candidate interval is a dotplot generated from pairwise sequence concordance in the mm9 genome assembly. Diagonal lines slanting down from left to right are duplications, while diagonal lines slanting up from left to right are inversions. Above the dotplot are arrows that show the four duplications (SD1-4) and inversion (I5) identified. Panel B is a phylogenetic tree that depicts the relationship between the duplications. The phylogenetic tree was generated using the CLUSTALW2 alignment software [71]. Also shown are the ten MegaMUGA markers used for the PCA analysis and their positions in relation to the segmental duplications. Shown in panel C are probe hybridization plots for two of these markers, UNC31159403 and XiD2 (all plots are provided in Figure S2). The axes represent hybridization intensities for probes tracking alternative alleles at each marker. The colors correspond to the different functional *Xce* alleles: gray *Xce^a^*; blue *Xce^b^*; red *Xce^e^*; green *Xce^c^*; yellow *Xce^d^*. Note that these plots do not agree with the expectations for standard biallelic variants. Typically biallelic variant plots show three distinct clusters representing homozygous A, homozygous B, or heterozygous A/B.

After phenotyping of the additional strains, the minimum candidate interval spans 176 kb and its size and relative position with respect to the *Xic* does not change in the latest mouse genome assembly (GrCm38/mm10). The final interval contains five protein coding genes, six pseudogenes, and three novel rRNAs. The G+C content is elevated compared to the X chromosome average (44% *versus* 39%, respectively [47]). Repeat masker [48] identified 50 LINEs and 60 SINEs as well as 194 other DNA features such as LTRs and regions of low complexity. However, the most dramatic feature is the presence of a set of tandem duplications and inversion (Figure 4A). The NCBI37/mm9 (and the GrCm38/mm10) reference assembly contains four tandem duplications and one inversion herein referred as segmental duplication (SD) 1 (99,909,337–99,942,773 bp), SD2 (99,940,942–99,961,388 bp), SD3 (99,959,575–100,013,166 bp), SD4 (100,013,346–100,035,061 bp), and inversion (I) 5 (100,040,370–100,084,982 bp) (Figure 4A). The average size of the duplications is 35 kb, the C+G content is 45%, and they typically span three genes, nine LINEs and 13 SINEs. The phylogenetic tree reveals that two pairs of duplications (SD1 and SD2 and SD3 and I5) are relatively recent events while duplication 4 is the oldest (Figure 4B). The topological arrangement of these SDs cannot be explained simply by a set of tandem duplications. In particular, the phylogentic origin, location and orientation of SD3, SD4 and I5 requires both an inversion and a deletion after the duplication event of their common ancestor (Figure 4B).

**Figure 5:**
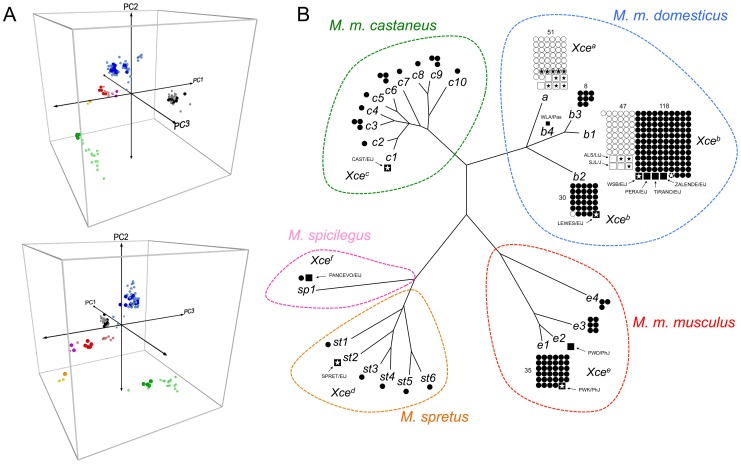
Natural history of *Xce*. Panel A shows a three-dimensional PCA plot based on hybridization intensity of ten MegaMUGA probes (Figure 4 and Table S10) within the refined *Xce* candidate interval. Mouse strains with known *Xce* alleles are shown as large spheres, while predicted mouse strains and wild-mice are shown as smaller spheres. Mouse samples are shaded according to *Xce* allele or *Xce* haplotype: Known *Xce^a^* allele, black; predicted *Xce^a^* allele, gray; known *Xce^b^* allele, blue; predicted *Xce^b^* allele, light blue; known *Xce^c^* allele, green; predicted *Xce^c^* allele, light green; known *Xce^d^* allele, orange; predicted *Xce^d^* allele, yellow; known *Xce^d^* allele, orange; predicted *Xce^d^* allele, yellow; known *Xce^e^* allele, red; predicted *Xce^e^* allele, pink; known *Xce^f^* allele, magenta. Panel B shows a phylogenetic tree based on 18 MDA SNP probes within the new *Xce* candidate interval. The topography of the tree accurately reflects the genetic relationship between the *Xce* alleles, however because of the limited number of SNP used to generate the tree and the ascertainment bias of the SNPs present on the MDA [40], [41], the tree is misleading with respect to the true genetic distance between *Xce* haplotypes (see Figure S4 for a more accurate representation of branch lengths). Open circles represent classical inbred strains with unknown *Xce* alleles; filled circles represent wild-derived or wild-caught mice with unknown *Xce* alleles; open squares represent classical inbred strains phenotyped for *Xce*; filled squares represent wild-derived strains with known *Xce* alleles. Strains with whole genome sequence data are shown with a star. We color coded the specific or subspecific origin of the candidate interval for the four major branches of the tree: red, *M. m. musculus*; blue, *M. m. domesticus*; green, *M. m. castaneus*, orange, *M. spretus*, pink, *Mus spicilegus* [53].

Because genotypes in segmental duplications are notoriously unreliable [40], [44], we investigated whether probes designed to track the duplications in the newly released MegaMUGA array (to be reported elsewhere) support our haplotype assignment and mapping conclusions. The MegaMUGA array was designed on Illumina's (San Diego, CA) Infinium BeadChips platform that consistently produces high signal-to-noise ratio compared to conventional hybridization based arrays as demonstrated by previous studies [49], [50]. These probes (Figure 4B, C and Table S5) consist of standard SNPs and probes with off target variants (VINOs) [51], [52] in addition to probes designed specifically to target the five duplications within the *Xce* candidate interval. Haplotype inference based on probe hybridization has been used successfully in other mouse populations such as the Collaborative Cross [45], [52]. We found a striking consistency between the haplotypes defined by nominal genotypes and the haplotypes based on principal component analysis (PCA) of probe intensities in the segmental duplications. In fact, MegaMUGA probe intensities perfectly partition all mouse inbred strains according to their experimentally defined *Xce* alleles. This is true not only for *Xce^a^* and *Xce^b^* carriers, but also for known *Xce^c^*, *Xce^d^*, *Xce^e^*, and *Xce^f^* carriers (Figure 5B). We extended this approach to analyze 110 genotyped samples with unknown *Xce* alleles (Figure 5A and Table S10). Samples with *M. m. domesticus* haplotypes in the candidate interval are partitioned into two groups corresponding to known carriers of *Xce^a^* and Xce*^b^* alleles, matching perfectly the results obtained by standard phylogenetic analysis. In addition, we found that wild-derived inbred strains as well as wild-caught mice with *M. spretus*, *M. spicilegus*, *M. m. castaneus* and *M. m. musculus* haplotypes cluster with the appropriate known carriers of an *Xce^d^*, *Xce^f^*, *Xce^c^*, and *Xce^b^*, respectively. We note that the probes used in the PCA do not share sequence similarity and they do not track homologous regions within the duplications and inversion. Finally, no single probe (nor pair of probes) is able to partition all samples according to *Xce* haplotype or functional allele. There are, however, certain probes that contribute to the partitioning of the *Xce* alleles more than others (highlighted in Figure 4B). These results indicate that no single probe can explain the *Xce* allelic series and that each probe does not track a different *Xce* allele.

Structural variation has been reported among inbred strains in the region encompassing the segmental duplications [53]. These structural variants are likely responsible for the difference in hybridization intensities and thus for the different haplotypes observed by PCA. These analyses strongly support the hypothesis that variation in the segmental duplications is associated with the five different functional *Xce* alleles.

### Phylogenetic analysis of the *Xce* candidate interval

To investigate the evolutionary history of the *Xce* locus, we generated phylogenetic trees based on genotype or sequence data (depending on availability) within the final minimum *Xce* candidate interval for 99 classical inbred strains, 66 wild-derived inbred strains and 124 wild-caught mice (Figure 5B and Table S6). This tree partitions these samples among five taxa, *M. spicilegus*, *M. spretus*, *M. m. castaneus*, *M. m. musculus* and *M. m. domesticus* that are consistent with previous studies [40], [41]. The *Xce* phenotype has been determined for at least one strain from each one of these taxa (Table S6). We found that each taxon (species or major subspecies) has a different functional *Xce* allele and there is no evidence of shared of alleles among taxa (Figure 5B). Skewed XCI is present in all crosses between wild-derived strains belonging to different taxa. In contrast, skewing is not present in crosses involving strains from the same taxon.

Within the *M. m. domesticus* subspecies we identified five haplotypes (*a*, *b1*, *b2*, *b3* and *b4*). The *a* haplotype is associated with *Xce^a^* while two haplotypes, *b1* and *b2* are associated with *Xce^b^*. The *b3* haplotype can be explained as recombination between a proximal *b2* and distal *b1* haplotype. The *b3* haplotype has been observed in either a small mouse population on the Farallon islands off the coast of San Francisco, CA, and in one wild-caught mouse from Barcelona, Spain. The *b4* haplotype appears to be a recombination between the *a* and *b1* haplotypes and is found only in the WLA/Pas strain that carries an ambiguous *Xce* allele.

Interestingly, there is an unequal distribution in the number and origin of *M. m. domesticus* stocks that carry each haplotype. For example, classical inbred strains are almost evenly divided among the *a* haplotype (*n* = 52) and the *b1* haplotype (*n* = 47) (Figure 5B). One classical inbred strain, CE/J carries the *b2* haplotype. CE/J has been reported to be an outlier among classical inbred strain because it has the smallest fraction of haplotype sharing genome wide with strains with WGS available [54].

In contrast, wild-derived and wild-caught *M. m. domesticus* mice exclusively carry the *b1*, *b2*, *b3* and *b4* haplotypes (Figure 5B). Note that we have determined experimentally the *Xce* allele for a wild derived representative of these two haplotypes. WSB/EiJ, PERA/EiJ, TIRANO/EiJ and ZALENDE/EiJ carry the *b1* haplotype and LEWES/EiJ carries the *b2* haplotype. All five wild-derived strains (WSB/EiJ, PERA/EiJ, TIRANO/EiJ, ZALENDE/EiJ and LEWES/EiJ) carry the *Xce^b^* allele.

We conclude that in natural populations *M. m. domesticus* mice predominantly (or exclusively) carry the *Xce^b^* allele. We further conclude that given its absence among 121 wild mice and wild-derived strains the *a* haplotype associated with the *Xce^a^* allele is likely a derived allele that arose concurrently with the domestication of fancy mice. Another possibility is that *Xce^a^* represents a rare allele in the wild (See Discussion, Figure 5B and Figure S4).

### Maternal inheritance of the strong *Xce* allele magnifies XCI skewing

**Figure 6:**
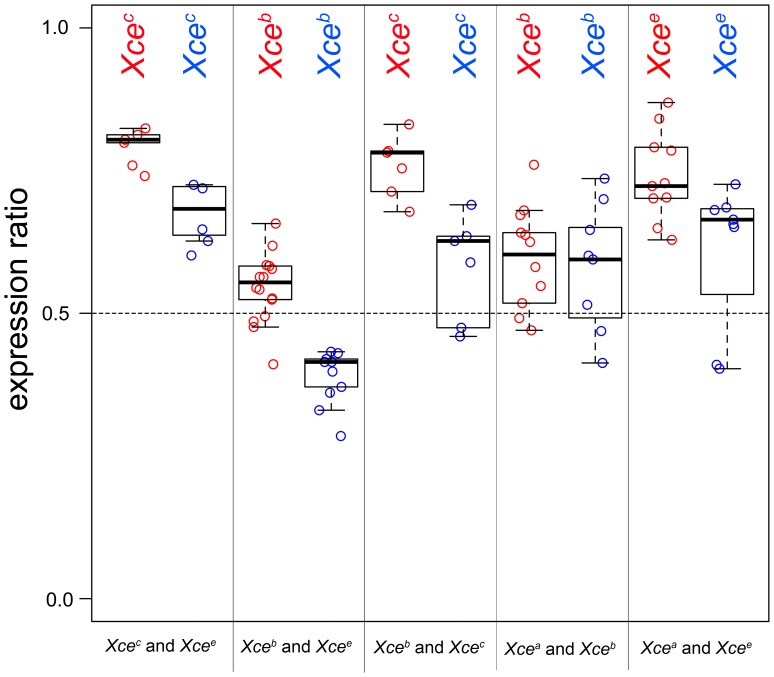
Maternal inheritance magnifies XCI skewing. Shown is allele-specific expression from reciprocal F1 *Xce* heterozygotes. The X-axis is partitioned according to *Xce* allele pairs. The Y-axis is the ratio of allele-specific expression from the X chromosome harboring the stronger *Xce* allele. Ratios were determined using either RNAseq or pyrosequencing.

Previous studies have shown that the parent-of-origin of the *Xce* allele can influence the skewing of XCI [23], [24], [33], [34]. To investigate this effect in our data set, we examined the XCI skewing in reciprocal F1 female hybrids (Table S3) and tested whether the effect of the parent-of-origin on X inactivation ratio was statistically significant. In order to increase the statistical power to detect parent-of-origin effects we aggregated crosses with the same combination of *Xce* alleles, doing so under the assumption that the parent-of-origin effects are substantially greater than putative effects of genetic background [34]. We found that the parent-of-origin effect was highly significant overall (*p* = 0.0023) and was consistent in its direction, magnifying XCI skewing in the F1 female hybrids inheriting the stronger *Xce* allele from their mothers (Figure 6). The magnitude of its effect varied between 18% (the X-inactivation proportion in (CAST/EiJxWSB/EiJ)F1 females minus that in (WSB/EiJxCAST/EiJ)F1 females) and 2% (WSB/EiJxA/J)F1 females minus (A/JxWSB/EiJ)F1 females), averaging 9% among all crosses where reciprocals were tested. We note that the parent-of-origin effect is observed independent of whether XCI measurement is based on pyrosequencing or RNAseq data. We found less support for the parent-of-origin effect on X inactivation skewing in reciprocal F1 females generated by crosses between the WSB/EiJ strain (*Xce^b^*) and *Xce^a^* allele carriers (Table S3).

Retrospective analysis of reported parent-of-origin effects is fully consistent with our hypothesis that maternal origin of a strong *Xce* allele magnifies the skewing (data not shown).

## Discussion

Recent advances in mouse genetic resources [40], [41] provide an opportunity to resolve unanswered biological questions. Our method for association mapping integrates historical phenotyping data with these new genetic resources enabling us to reduce rapidly existing candidate intervals to a size amenable to mechanistic studies. This method is similar to approaches to identify candidate genes within candidate intervals reported previously [55], [56]. The method guides subsequent experiments by identifying additional mouse strains that could reduce the candidate interval through informative historical recombinations. Moreover, our comparative analysis of different subspecies of mouse provides unique insight into the evolutionary history of the locus that is key to explaining its allelic series [40].

The validity of our approach relies on the fulfillment of several assumptions. These include the requirement that the locus under study explains a large fraction of the genetic variance and its action to be largely independent of other loci; that the causative mutation(s) for each functional allele has arisen once during evolutionary history; and that the genetic markers used in the analysis reflect the true haplotype diversity in the entire candidate interval.

In our mapping of the *Xce* locus, fulfillment of the first assumption of a large genetic effect relies on 40 years of evidence that support the existence of a single major locus on the X chromosome near *Xic* that influence XCI choice [17], [19], [20], [21], [22], [23], [26], [57], [58]. Note that these studies arrive at the same conclusion regardless of the combination of *Xce* alleles (*Xce^a^*, *Xce^b^* and *Xce^c^*) used in each particular study. Although parent-of-origin and autosomal effects have been reported, the consensus is that their contribution to XCI skewing variation is small compared with that of *Xce* [23], [26], [34]. The need to fulfill the second assumption, that each allele arose once, guided the decision to restrict our initial association mapping analysis to classical inbred strains only, since the probability of multiple recurring mutations are extremely low based on their history [40], [41], [54]. Lastly, fulfilling the third assumption, we have previously shown that the marker density in MDA is sufficient to accurately reflect the underlying haplotype diversity genome wide and in particular in regions with lower levels of recombination such as the X chromosome [40], [41], [54].

We have shown that this approach was effective at rapidly reducing the *Xce* candidate interval 10-fold and that it may prove useful to map other genetic traits of interest provided that they meet the above listed criteria. In fact, *Xce* is a particularly difficult test case because of complexity of the XCI process and the reduced recombination rate on the X chromosome.

We tailored our experimental design to anticipate the challenges of phenotyping mouse strains with unknown *Xce* alleles. First, the functional allele in a strain with an unknown *Xce* allele can be determined only by generating heterozygous females with known *Xce* alleles and then determining the ratio of XCI in the heterozygous progeny. The precision in identifying the unknown allele increases with the number of different alleles to which it is paired in the experimental F1 hybrids. We, therefore, crossed each strain with an unknown *Xce* allele to at least two strains with known and different *Xce* alleles.

To estimate mean XCI skewing accurately, we phenotyped multiple females per cross. Moreover, for most females, we measured XCI skewing in at least three different tissues that roughly represent the three germ layers, brain (ectoderm), liver (endoderm) and kidney (mesoderm). Our results confirm previous reports that mean XCI skewing is similar between different tissues [25], [35], [59], [60]. We do, however, observe differences in the variance of XCI skewing between different tissues (brain ±6% kidney ±7.5%, and liver ±8.2%). From a practical standpoint, whole brain had the smallest variance and thus would require fewer animals to accurately determine mean XCI skewing.

It is appropriate to use gene expression to measure the proportion of cells using the maternal *versus* paternal X chromosomes. However, expression at single genes can be misleading because of measurement bias or allelic imbalance independent of XCI choice such as *cis-*acting regulatory variants or XCI escape. To mitigate these potential issues, we measured multiple X-linked genes using pyrosequencing and/or RNAseq. By combining multiple gene measurements, we can better estimate the mean XCI skewing. Both technologies simultaneously measure maternal and paternal expression, reducing the concern of parent-specific measurement bias.

Despite our thoroughness, we could not conclusively assign an *Xce* allele to the WLA/Pas strain, although we can exclude both *Xce^c^* and *Xce^d^*. A possible reason for this is that in all crosses involving WLA/Pas the *Xce^WLA/Pas^* allele was inherited through the paternal germline and in the absence of reciprocal crosses the parent-of-origin can potentially complicate *Xce* allele calling. A second, and more interesting explanation is that WLA/Pas has a *b4* haplotype that appears to be *a/b1* recombinant whose breakpoints fall within the SD4 in the candidate interval (see below and Figure 4).

Although only a small number of readily available mouse strains carry *M. m. castaneus* or *M. m. musculus* haplotypes, a previous study measured XCI skewing in reciprocal F1 hybrids between PWD/PhJ and AKR/J [61]. This study reported that PWD/PhJ has an *Xce* allele that is weaker than *Xce^b^*. This result matches our conclusion that PWK/PhJ, a closely related wild-derived inbred strain [40], carries the *Xce^e^* allele. Furthermore, we conclude that *M. m. musculus* do not carry the *Xce^c^* allele as reported in a congenic mouse line believed to be of *M. m. musculus* origin within the *Xce* candidate interval [59].

Our conclusion that the structural variants in the duplications within the candidate interval are likely to be responsible for the different *Xce* alleles provides simple and satisfactory answers to questions such as the presence of the allelic series, the overdominant nature and mechanism of action of *Xce*, and the evolutionary origin of the interspecific differences for XCI choice. Copy number variation within a region with complex segmental duplications and inversions can explain the large number (six alleles described so far in *Mus*) and different strength of the alleles at *Xce*. For example, the different strength of *Xce* alleles can be attributed to the number of copies of a binding site for a transfactor that is critical for the initiation of XCI [28], [29], [30], [31], [32].

One of the conclusions of our study is that each one of the five taxa (species or major subspecies) analyzed for XCI choice in *Mus* has a different functional allele and that there is no evidence of shared alleles between them. The rate of mutation for CNV at segmental duplicated regions fits well with the observed functional diversity at *Xce*. Given that unequal recombination is thought to be the primary process generating CNVs, it is noteworthy that two of the haplotypes reported here (*b3* and *b4*) involve crossing over within the duplications. In fact, we observe an apparently correct heterozygous call at SNP rs29082017 in two males with the *b3* haplotype. Given that males cannot be true heterozygotes for X linked markers, the result strongly suggests that an unequal crossing over has generated a new haplotype with paralogous variation. Resequencing the candidate interval in these strains should provide important information on the relationship between CNVs and functional *Xce* alleles.

It is striking that each species and subspecies examined thus far has a different functional allele. Furthermore, in the six wild-derived *M. m. domesticus* mouse strains phenotyped in this study, we do not find the occurrence of multiple functional alleles. We conclude that in *M. m. domesticus*, *Xce^b^* is the prevalent allele and other functional alleles are either rare or absent. The broad geographic origin of the wild-derived strains analyzed here strongly support this conclusion (Table S6). The only apparent exception to this rule is the presence of two functional alleles in classical inbred strains, *Xce^a^* and *Xce^b^*. That said, it is likely that *Xce^b^* is the ancestral allele within the *domesticus* subspecies and *Xce^a^* is a new, derived allele that originated early during the domestication of fancy mice. However, the phylogenetic tree shown in Figure 5B reveals deep branching between *Xce^a^* and *Xce^b^* haplotypes that at first glance suggests that both are old alleles. Upon further investigation, there is evidence that the deep branching observed in Figure 5B may be an artifact generated by genotyping and alignment problems in regions with segmental duplications (i.e., the apparent SNP are paralogous variants rather that allelic ones). Figure S4 provides evidence in favor of this later scenario as the deep branching disappears immediately proximal (Figure S4A) and distal (Figure S4C) to the duplicated regions. Furthermore, there is a dramatic increase in the density of heterozygous calls in the WGS data for inbred strains that overlaps the region of segmental duplications (Figure S4D).

The phylogenetic analysis also provides an explanation for the apparent differences in the genetics of XCI choice between mouse and humans. Mouse geneticists were able to find evidence of genetic control of XCI because they used mice derived from multiple taxa and because *Xce^a^* and *Xce^b^* are equally represented among classical laboratory inbred strains. In fact, were we to have studied only wild-derived or wild mice of *M. m. domesticus* origin, we would very likely have concluded that XCI choice is not under the control of a X chromosome linked locus. We speculate that this is probably the situation in humans too, but note that this conclusion would be due to a lack of functional variation at the *Xce* locus and not proof of the absence of a locus controlling XCI choice.

We conclude that *Xce* is the major determinant of primary XCI choice and maps 500 kb proximal to key components of the murine *Xic* (*Xist*, *Tsix* and *Xite*). Our results are compatible with the general conclusions reached by Thorvaldenson and coworkers (2012). Nonetheless a direct comparison of both studies is difficult. Thorvenson and colleagues (2012) used only two functional alleles, *Xce^a^* and *Xce^c^* from highly divergent mouse strains to map roughly X-linked regions influencing XCI choice. They found that all their crosses, regardless of heterozygosity within the Chadwick interval, there is some degree of skewing in favor of the 129S1/SvlmJ and CAST/EiJ recombinant chromosome X. This led to the conclusion that multiple X-linked loci influence XCI choice. Although we provide strong evidence that the *Xce* allelic series is due to structural variation in the *Xce* candidate interval, we cannot exclude that a selected few SNPs within the Chadwick interval may also contribute to XCI choice. There are 14 SNPs distal to the *Xce* interval reported here with consistent SDPs in *M. m. domesticus* after the incorporation of the four strains with *M. m. domesticus* phenotyped. None of these SNPs individually can explain the allelic series and no simple combination of them within a single gene can be directly tied to the phenotype. On the other hand our reciprocal crosses between ALS/LtJ and C57BL/6J agree with Thorvenson's hypothesis that additional loci may have an effect in XCI choice as we find that the parent-of-origin effect is present despite homozygosity at the *Xce* locus (Figure S3). Both studies strongly predict the presence of an additional X-linked locus (or loci) controlling the parent-of-origin effect.

The genetic analysis of the *Xce* locus presented in this study sets the stage for the molecular characterization of *Xce*. However, the most direct experiments will require access to the cells and biological material of the critical window at which XCI choice is made either by *in vivo* or *ex vivo* using ES cell lines.

## Materials and Methods

### Mouse breeding and tissue isolation

Mice from nine inbred strains (129S1/SvlmJ, A/J, ALS/LtJ, C57BL/6J, CAST/EiJ, LEWES/EiJ, PWK/EiJ, SJL/J, and WSB/EiJ,) were originally obtained from the Jackson Laboratory (Bar Harbor, ME). Mice of the WLA/Pas strain were generously provided by Xavier Montagutelli from the Pasteur Institute (Paris, FR). Mice were bred at UNC-Chapel Hill for multiple generations and interbred to generate F1 hybrids. Litters of F1 mouse pups were sacrificed within 24 hours after birth. We harvested whole brain, whole liver, right kidney, tail and a forepaw (for sexing, [62]). Tissues were infused with RNAlater (Qiagen) and frozen at −80°C to preserve RNA integrity until extraction. Whole brain was isolated from mouse pups derived from crosses (DDKxC57BL/6J)F1 X PANCEVO/EiJ, (C57BL/6J X DDK)F1 X TIRANO/Ei and (C57BL/6J X DDK)F1 X ZALENDE/Ei [63] and (C57BL/6J X PERA)F1 X C57BL/6J [64]. These mouse crosses were generated for previous studies and reported elsewhere. All mice were treated according to the recommendations of the Institutional Animal Care and Use Committee (IACUC) of the University of North Carolina at Chapel Hill.

### Ethics statement

All mice were treated according to the recommendations of the Institutional Animal Care and Use Committee (IACUC) of the University of North Carolina at Chapel Hill. To minimize the number of animals bred to determine the X inactivation pattern associated with a given *Xce* genotype we used whole brain from samples generated an stored as part of a previous study from crosses (DDKxC57BL/6J)F1 X PANCEVO/EiJ, (C57BL/6J X DDK)F1 X TIRANO/EiJ, (C57BL/6J X DDK)F1 X ZALENDE/Ei and (C57BL/6J X PERA)F1 X C57BL/6J. These mouse crosses have been reported elsewhere. For samples generated in this study, mice were bred at UNC-Chapel Hill to generate the required F1 hybrid females. Litters of F1 mouse pups were sacrificed within 24 hours after birth using an approved protocol that minimizes pain and suffering of newborn pups.

### Genotypes

Mouse genotypes were acquired from recent studies that employed next-generation sequencing [41], [53] and high-density genotyping array technology [40], [44]. Tables S1, S4, and S6 provide a list of all mice (inbred and wild-caught) and the origin of the genotype information. As an initial filtering step, heterozygous and low-confidence genotyping calls were removed from the data set. Heterozygosity within the *Xce* candidate interval was determined in F2 mouse pups using microsatellite marker *DXMit16* (∼99.3 Mb) [65]. Genomic DNA was amplified according to previously reported conditions with the exception of a fluorescent label covalently bound to one *DXMit16* primer (6-FAM-5′-CTgCAATgCCTgCTgTTTTA-3′). 0.5 µl of amplified products were resuspended in 9.0 µl of HIDI formamide (Life Technologies) and 0.5 µl of LIZ1200 sizing ladder (Life Technologies). Samples were run on the ABI 3730xl DNA analyzer using long-run fragment analysis conditions. Traces were analyzed with ABI PeakScanner software.

### Association mapping

At each diallelic variant within the Chadwick interval, we represented the C57BL/6J (or C57BL/6JN) allele as zero and all other strains with the same genotype as zero. Strains with the alternative allele are represented with the number one. We then generated strain distribution patterns for each variant as a series of ones and zeros for the strains in the following order: 129S1/SvlmJ, A/J, BALB/cByJ, C3H/HeJ, CBA/J, DDK/Pas, C57L/J, DBA/1J, DBA/2J, and AKR/J (Table S1). We classified an SDP as completely consistent when all *Xce^a^* allele carriers are ones (share the same allele) and all *Xce^b^* allele carriers are zeros (share the same allele as C57BL/6J) (Tables S1 and S4). We defined an inconsistent SDP when one or more *Xce^a^* strain(s) are zeros and one or more *Xce^b^* strain(s) are ones (*i.e.* A/J, 129S1/SvlmJ, BALB/cByJ, C3H/HeJ, CBA/J, AKR/J opposite to DDK, C57BL/6J, DBA/1J, DBA/2J) (Tables S1 and S4). Lastly, we defined a diallelic variant as partially consistent when one or more *Xce^a^* strain(s) are zeros or one or more *Xce^b^* strain(s) are ones (Tables S1 and S4).

### Measuring allelic imbalance in F1 female hybrids

mRNA was extracted from tissues of F1 mice using an automated bead-based capture technology (Maxwell 16 LEV TotalRNA Kits, Promega). Purified mRNA was checked for quality and quantity using a Nanodrop spectrophotometer (Thermo Scientific). For each sample, mRNA was retrotranscribed (SuperScript III, Life Technologies) to produce cDNA. We designed primers (Table S7) to capture expression SNPs (Table S8) within X-linked genes to serve as surrogates for maternal and paternal XCI status. In individual reactions, we amplified 1 µl of cDNA in a final volume of 30 µl for 35 cycles (See Table S7 for PCR cycling conditions). One primer for each assay was biotinylated in order to immobilize and purify the amplified products using streptavidin beads (GE Healthcare) according to the manufacturer's protocol (Qiagen). We used Pyrosequencing technology to measure the proportion of maternal and paternal X-linked gene expression simultaneously. Pyrosequencing quantitatively measures, in real-time, the release of pyrophosphate as a result of nucleotide incorporation during the polymerase chain reaction [66]. Purified, single-stranded amplicons were primed for pyrosequencing using gene-specific primers (Table S7) and pyrosequenced using the PyroMark Q96 MD instrument (Qiagen) and PyroMark Gold Q96 Reagents (Qiagen) according to manufacturer's protocols. Allelic proportions were determined by the quantitative analysis option of the PyroMark Q96 MD Software. Raw results are show in Table S9.

### RNAseq analysis

RNAseq data used in this study is reported elsewhere (Crowley et al., 2013, unpublished). Briefly, we generated cDNA libraries (Illumina (San Diego, CA) TruSeq RNA Sample Preparation Kit v2) from whole brain mRNA of female reciprocal F1 hybrids between CAST/EiJ, PWK/PhJ, and WSB/EiJ. Using the Illumina HiSeq 2000 instrument, we sequenced 100 bp paired end reads (2×100). For each F1 hybrid, we mapped 100 bp paired-end RNAseq reads to pseudogenomes of each parent (CAST/EiJ, PWK/PhJ and WSB/EiJ) using TopHat. Pseudogenomes are approximations of CAST/EiJ, PWK/PhJ and WSB/EiJ strain genomes constructed by incorporating all known SNPs and indels into the C57BL/6 genome (mm9) [67]. We allowed two mismatches total per 100 bp read. For each read, we annotated the number of maternal and paternal alleles (using SNPs and indels). XCI ratios were determined by counting the number of maternal reads versus the number of paternal reads. To measure XCI ratios, we selected 10 X-linked genes that are distributed across the X chromosome (*Wdr13*, *Atp6ap2*, *Usp9x*, *Cask*, *Cd99l2*, *Idh3g*, *Dlg3*, *Zcchc18*, *Tsc22d3*, *Iqsec2*). For each gene, we selected two informative SNPs between PWK, CAST, and WSB so that at least five of the ten genes were informative for a given F1 hybrid. For each informative SNP, we counted allele-specific reads to determine XCI ratios. Results are summarized in Table S9.

### Statistical model for cross-specific X-inactivation ratios

Pyrosequencing and RNAseq provided estimates of the X-inactivation ratios obtaining for particular genes in specific tissues in particular individuals. In order to infer X-inactivation ratios pertaining to individual mice and to the crosses that generated them, we developed a hierarchical Bayesian model linking the observed experimental measurements to a structured set of higher order parameters. These parameters reflected not only the stochastic relationships between measurements, individuals and crosses, but also between different sources of experimental variation. Let 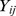 be the measured X-inactivation proportion from pyrosequencing or RNAseq in the 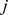
th gene-tissue combination of the 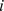
th mouse, and let 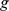 be the F1 cross to which mouse 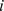 belongs, where for instance, crosses (129S1/SvlmJxPWK/PhJ)F1 and (PWK/PhJx129S1/SvlmJ)F1 are distinct. We first model a latent variable 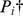 representing the X-inactivation proportion inherent to the individual mouse 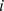 as if arising from a beta distribution
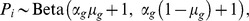
with cross-specific mean governed by 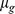 and cross-specific variance proportional to 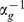. This individual-specific parameter 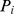 then forms the basis of a further beta distribution, which models tissue-gene specific measurements 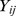 as if generated by
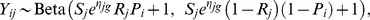
where 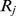 and 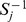 are the bias and variance introduced by tissue-gene combination 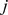, and where 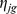 allows for cross-specific variance in X-inactivation. All higher order parameters are themselves modeled in loosely-specified grouped hierarchies based realistic but vague priors (as in, eg [68]). This hierarchical structure allows information and uncertainty to propagate within and between parameters, and results in improved estimation through shrinkage (see, eg, [69]). We obtain posterior distributions for all parameters, including those representing unobserved data, using Markov Chain Monte Carlo (MCMC). Marginal posterior probability densities are computed for 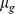 parameters for crosses between mice with unknown *Xce* alleles using information from mice with known alleles. The 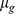 posterior density that includes the most support for 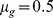 is taken as the most plausible candidate for having *Xce* allele shared by the unknown strain. In general, posteriors for 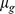 concentrated near 0.5 are more consistent with there being a shared allele between maternal and paternal pairs, whereas posterior densities shifted from 0.5 suggest that the *Xce* is different.

### Significance test of parent-of-origin effects

The statistical significance of parent-of-origin effects was determined by permutation. We first estimated the difference in specimen-level X-inactivation, 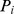, between genetically matched individuals of reciprocal parentage and unequal *Xce* alleles, and used this estimate as our test statistic. We then repeated this estimation under 10000 shuffles of the parent-of-origin labels in order to generate a null distribution of the test statistic, and thereby estimate a p-value for the parent-of-origin effect in the real data.

### Principal Component Analysis (PCA)

For each sample, we constructed a vector of Illumina probe intensities of MegaMUGA markers within the refined *Xce* candidate interval (Table S10). We then performed principal component analysis on these vectors and report the projection of each sample onto the first three principal components.

### Phylogenetic analysis

For each inbred strain and wild-caught mouse, we assigned the subspecific origin of the Chadwick and new *Xce* candidate interval based on diagnostic alleles from SNP and VINO calls 40,51. We then built DNA distance, maximum likelihood, and DNA parsimony phylogenetic trees (PHYLIP (Phylogeny Inference Package) [70]) based on all variation within the candidate interval. No major differences were observed between analysis types, so we chose maximum likelihood with 100 bootstraps to represent the phylogenetic relationship between mice in Figure 5B.

## Supporting Information

Figure S1 Strain Distribution Patterns (SDP). This Figure depicts how the patterns of strain genotypes were classified as consistent, inconsistent or incompletely consistent with the *Xce* phenotypes. SNPs or indels that partition the strains according to their *Xce^a^* and *Xce^b^* phenotype were classified as “consistent” and represented as a black (or blue) tick mark in Figure 2B. SNPs or indels that are shared by both *Xce^a^* and *Xce^b^* strains were classified as an SDP that is “inconsistent” with the *Xce* phenotypes and represented as a red tick mark in Figure 2B. Lastly, A SNP or indel that is partially consistent but not inconsistent with the *Xce* phenotypes was classified as “partially consistent” and represented with a gray tick mark in Figure 2B.
(TIF)

Figure S2 MegaMUGA probe plots. Each of the ten panels is a hybridization plot of an individual MegaMUGA probe targeting the *Xce* candidate interval. As described in Figure 4, the axes represent hybridization intensities for probes tracking alternative alleles at each marker. The colors correspond to eight biological replicates of the eight founder inbred strains of the Collaborative Cross. Yellow A/J; black C57BL/6J; pink 129S1/SvlmJ; blue NOD/ShiLtJ; light blue NZO/HiLtJ; green CAST/EiJ; red PWK/PhJ, and purple WSB/EiJ. Samples in gray represent 300 control DNAs.
(TIF)

Figure S3 Allelic imbalance in additional strains characterized. Shown in panel A are scatter plots and posterior mean and confidence intervals for additional strains phenotyped in this study. Shown in panel B is the posterior distributions of the phenotyping data in panel A.
(TIF)

Figure S4 Phylogenetic analysis of the *Xce* and flanking intervals using whole genome sequence data. Shown are DNA distance trees based on whole genome sequence data [41], [53] within the corresponding intervals. Panel D shows the SNP density (solid line) and heterozygosity (dashed lined) within the candidate (Panel B) and flanking intervals (Panels A and C).
(TIF)

Table S1 Genotype data in the Chadwick interval for strains with previously known *Xce* allele. This table summarizes consistent, inconsistent and partially consistent SDPs for inbred mouse strains with previously known *Xce* alleles. The data includes MDA and Sanger sequencing data.
(XLSX)

Table S2 Justification of selected inbred strains. This table lists the justification for selecting each strain and summarizes the number of F1 females phenotyped for each inbred strain with an unknown *Xce* allele.
(XLSX)

Table S3 Summary of crosses. This table summarizes all strains and crosses phenotyped in this study, their corresponding *Xce* alleles, and the molecular method used to measure allele-specific expression. In addition, listed are the posterior mean, median and confidence intervals determined by the Bayesian hierarchical model.
(XLSX)

Table S4 Genotype data in the Chadwick interval for strains with known *Xce* allele. This table summarizes consistent, inconsistent and partially consistent SDPs for inbred mouse strains with previously known *Xce* alleles combined with mouse strains phenotyped in this study.
(XLSX)

Table S5 MegaMUGA probe information. Summarized in this table are the 10 MegaMUGA probes used in the principal component analysis. Shown are the probe names, sequences and ranking according to how much each probe contributes to each principal component.
(CSV)

Table S6 List of all mouse samples. This table lists each mouse samples used in this study (total of 327). We annotated haplotypes based on its association with mouse strains with known *Xce* alleles; we assigned the subspecific origin of the *Xce* candidate interval, whether the mouse is a classical inbred [65], wild-derived inbred, or wild-caught; and we assigned each classical strain to a subclass [42] and each wild-derived or wild-caught to a geographic origin. For each mouse sample, we list the haplotype based on 18 MDA genotypes, the name of the haplotype (*i.e. a*, *b1*, *b2*, etc), The letter “V” stands for variable intensity oligonucleotide (VINO) [51], the letter “H” stands for heterozygous, and the letter “N” stands for no call.
(XLSX)

Table S7 Primers and conditions for pyrosequencing assays. Primer sequences and annealing temperatures for primer pairs are shown. For amplification prior to pyrosequencing, a universal PCR protocol was used but the annealing temperature was tailored specifically to each primer pair.
(XLSX)

Table S8 Pyrosequencing expression assay allele information. The table shows the mouse strains phenotyped and their genotype for each pyrosequencing assay used. Strains without genotype information are labeled “N/D.”
(XLSX)

Table S9 Pyrosequencing and RNAseq raw data. This matrix shows the fraction of maternal expression generated from pyrosequencing and RNAseq of mouse pups. Each row represents an individual mouse and each column represents a gene measurement. NA is used to show missing data.
(CSV)

Table S10 PCA results. Shown are the first three principal components used to generate Figure 5A for each mouse sample.
(CSV)
