# Supplementary figures and images for: Genetic Architecture of Skewed X Inactivation in the Laboratory Mouse
Source: PLoS Genet. 2013 Oct 3;9(10):e1003853. doi: 10.1371/journal.pgen.1003853 (PMC3789830; doi:10.1371/journal.pgen.1003853)

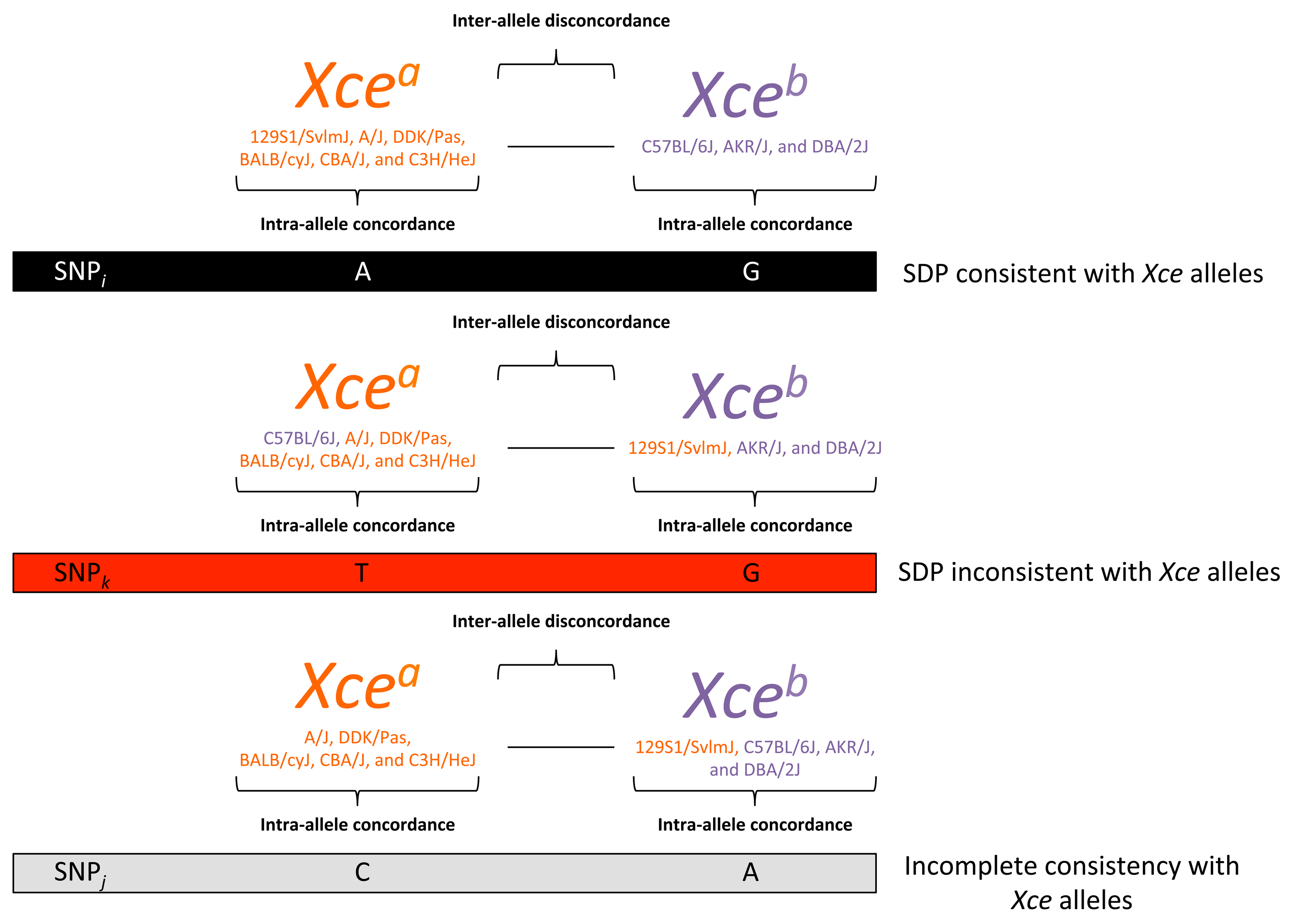

Supplement: Figure S1 — Strain Distribution Patterns (SDP). This Figure depicts how the patterns of strain genotypes were classified as consistent, inconsistent or incompletely consistent with the Xce phenotypes. SNPs or indels that partition the strains according to their Xcea and Xceb phenotype were classified as “consistent” and represented as a black (or blue) tick mark in Figure 2B. SNPs or indels that are shared by both Xcea and Xceb strains were classified as an SDP that is “inconsistent” with the Xce phenotypes and represented as a red tick mark in Figure 2B. Lastly, A SNP or indel that is partially consistent but not inconsistent with the Xce phenotypes was classified as “partially consistent” and represented with a gray tick mark in Figure 2B. (TIF) [file pgen.1003853.s001.tif]

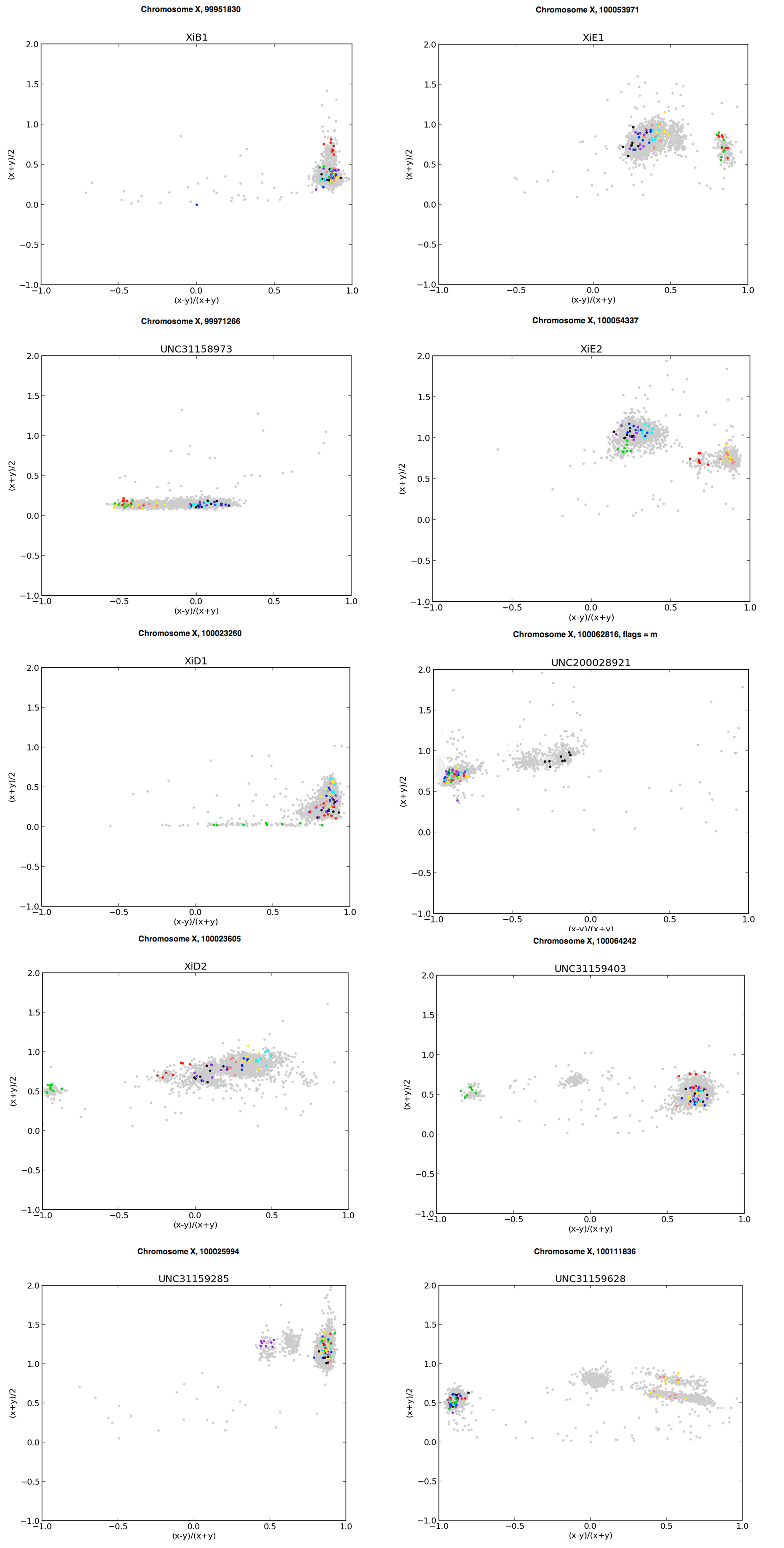

Supplement: Figure S2 — MegaMUGA probe plots. Each of the ten panels is a hybridization plot of an individual MegaMUGA probe targeting the Xce candidate interval. As described in Figure 4, the axes represent hybridization intensities for probes tracking alternative alleles at each marker. The colors correspond to eight biological replicates of the eight founder inbred strains of the Collaborative Cross. Yellow A/J; black C57BL/6J; pink 129S1/SvlmJ; blue NOD/ShiLtJ; light blue NZO/HiLtJ; green CAST/EiJ; red PWK/PhJ, and purple WSB/EiJ. Samples in gray represent 300 control DNAs. (TIF) [file pgen.1003853.s002.tif]

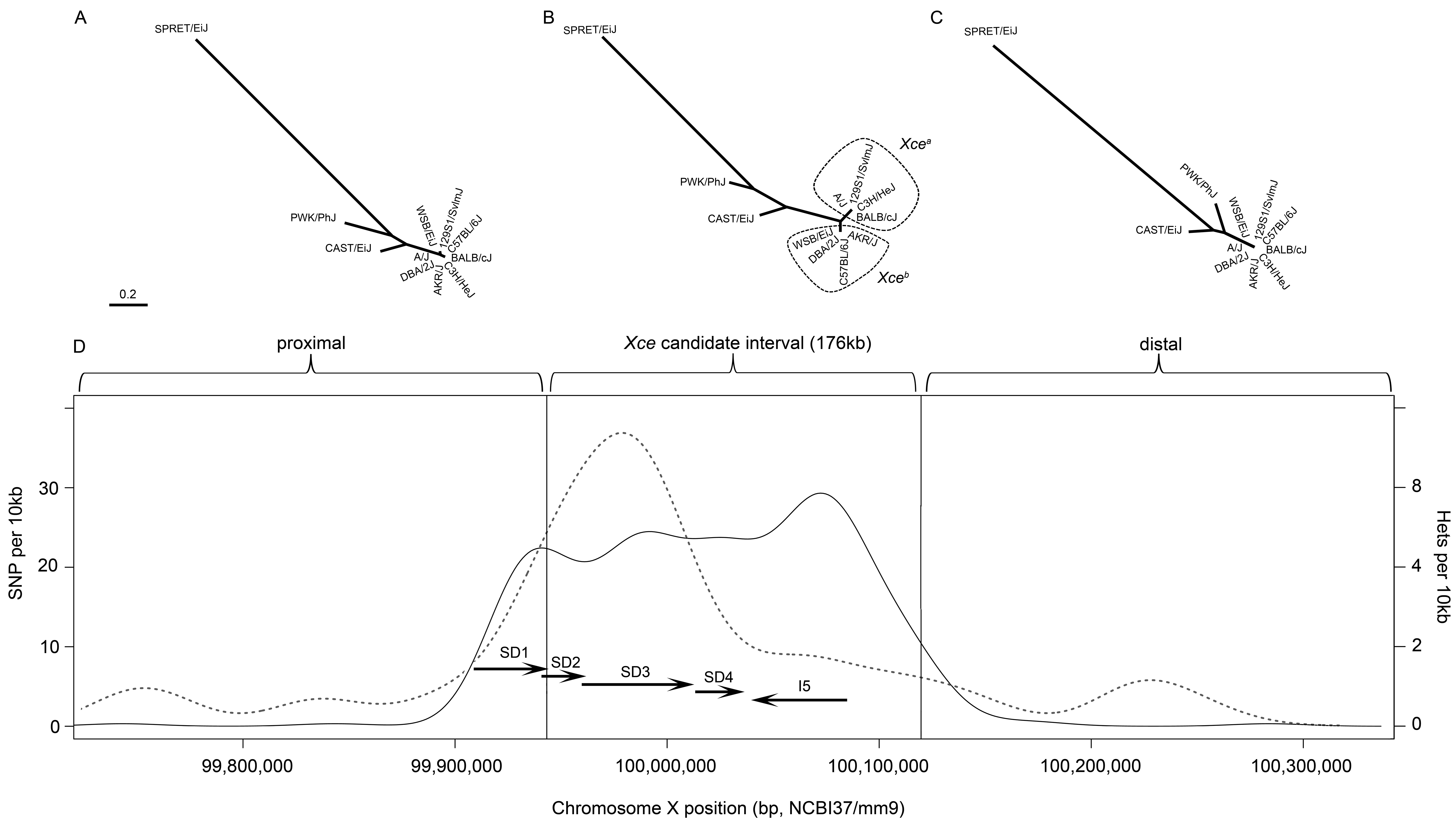

Supplement: Figure S4 — Phylogenetic analysis of the Xce and flanking intervals using whole genome sequence data. Shown are DNA distance trees based on whole genome sequence data [41], [53] within the corresponding intervals. Panel D shows the SNP density (solid line) and heterozygosity (dashed lined) within the candidate (Panel B) and flanking intervals (Panels A and C). (TIF) [file pgen.1003853.s004.tif]
